# Scurvy, abnormal MRI, and gelatinous bone marrow in an adolescent with avoidant restrictive food intake disorder

**DOI:** 10.1186/s40337-023-00770-7

**Published:** 2023-03-20

**Authors:** Ginny Claire Kim, Asha M. Davidson, Rebecca M. Beyda, Mona A. Eissa

**Affiliations:** University of Texas Health Science Center, 6431 Fannin, MSB 3.150, Houston, TX 77030 USA

**Keywords:** Scurvy, Vitamin C deficiency, Avoidant restrictive food intake disorder, ARFID, Gelatinous transformation of bone marrow, GBM

## Abstract

**Background:**

Although medical literature describes pediatric scurvy as “rare”, a growing number of case reports suggests otherwise. Patients often undergo costly and unnecessary workup due to unfamiliarity with the presentation of scurvy. This case report further supports the small yet growing literature documenting scurvy and its manifestations in patients with eating disorders.

**Case presentation:**

A 15-year-old female presented to the emergency department with bilateral knee and ankle swelling and pain in the setting of chronic lower limb rash and BMI of 16.3. For years, she had restricted her diet to carbohydrates. Exam showed perifollicular petechial hemorrhagic rash with corkscrew hairs, knee edema, ankle edema with restricted range of motion, and antalgic gait. She was admitted for severe malnutrition from avoidant restrictive food intake disorder. Her hospital course was complicated by recurrent normocytic anemia and fever. Hematology workup revealed anemia from iron deficiency, vitamin K deficiency, and anemia of chronic disease. Rheumatology workup was negative. MRI findings showed dark T1 and bright T2 signals and were read as consistent with leukemia/lymphoma, chronic multifocal osteomyelitis, or Langerhans cell histiocytosis. However, bone marrow biopsy showed gelatinous transformation secondary to malnutrition. She was treated with vitamin C and a nutrition plan and her symptoms improved.

**Conclusions:**

Although this patient had common manifestations of scurvy, including perifollicular petechial hemorrhagic rash, joint effusions, anemia, and recurrent fevers, she still underwent an extensive workup. Clinicians should be aware that scurvy can present with multiple symptoms that mimic infectious, rheumatic, oncologic and hematological disease. Clinicians should have a high index of suspicion for scurvy in patients with malnutrition and eating disorders.

## Background

Currently, scurvy is thought of as “rare” and irrelevant to patients in developed countries. Many providers are unfamiliar with the presentation of scurvy. We present a case of an adolescent with an eating disorder who, despite having classic features of scurvy, underwent a lengthy and extensive workup. Her case is not isolated, and many patients have received similar workups. In a recent case series, authors found over 50 pediatric cases of scurvy over the past 20 years [1]. Despite the growing amount of literature, awareness about scurvy remains limited.

## Case presentation

**Fig. 1:**
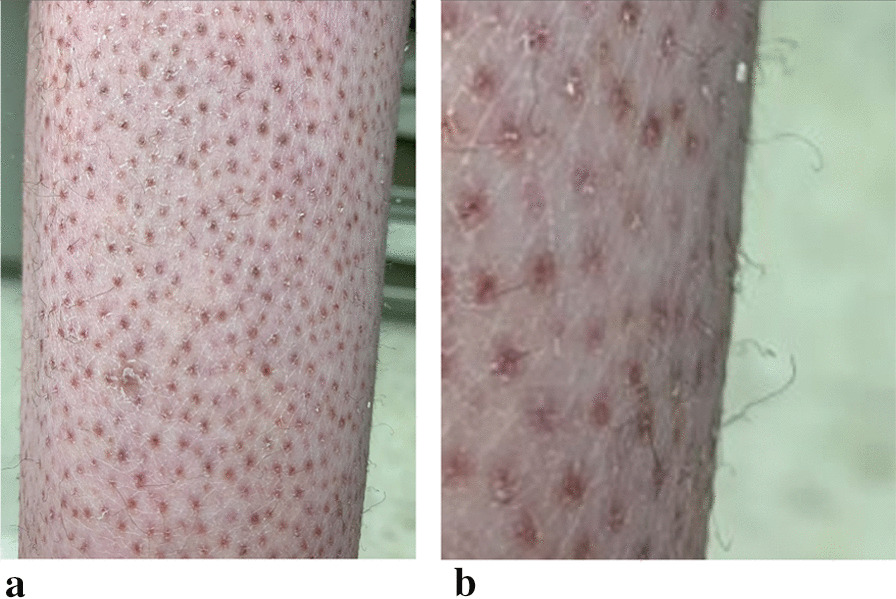
Photos of the patient’s perifollicular petechial hemorrhagic rash with corkscrew hairs. **a** The rash on the legs which had been present for seven years. **b** The corkscrew hairs

**Fig. 2:**
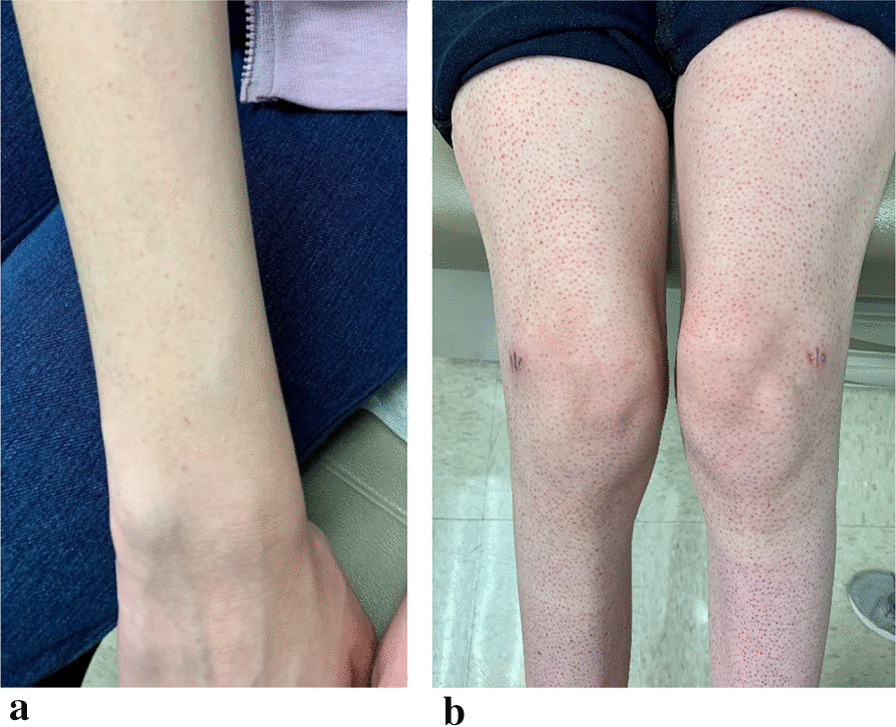
**a** Development of faint rash on forearms. **b** The bilateral knee swelling and leg rash with healed biopsy incisions

A 15-year-old female with history of mild depression presented to the emergency department with acutely worsening bilateral knee pain, rash, easy bruising, and fatigue. She noticed the bilateral leg rash seven years ago (which she kept hidden from her parents). In the preceding two months, a rash formed on her wrists, and her knees and ankles swelled painfully. She also had ten months of amenorrhea. Exam showed perifollicular petechial hemorrhagic rash with corkscrew hairs that extended from her ankles superiorly and multiple ecchymosis (Figs. 1, 2). She had bilateral knee and ankle edema without overlying warmth or rubor (Fig. 2b). Additionally, she had limited ankle range of motion with significant restriction of plantar dorsiflexion and plantarflexion as well as an antalgic gait. She weighed 48.6 kg (34th percentile) with height 172.7 cm (95th percentile) and BMI 16.3 (5th percentile). She had tachycardia with abnormal orthostatic vital signs. History revealed a restricted diet of bread, cereal, chips, and butter. We diagnosed her with avoidant restrictive food intake disorder (ARFID), and she was admitted for severe malnutrition.

**Fig. 3:**
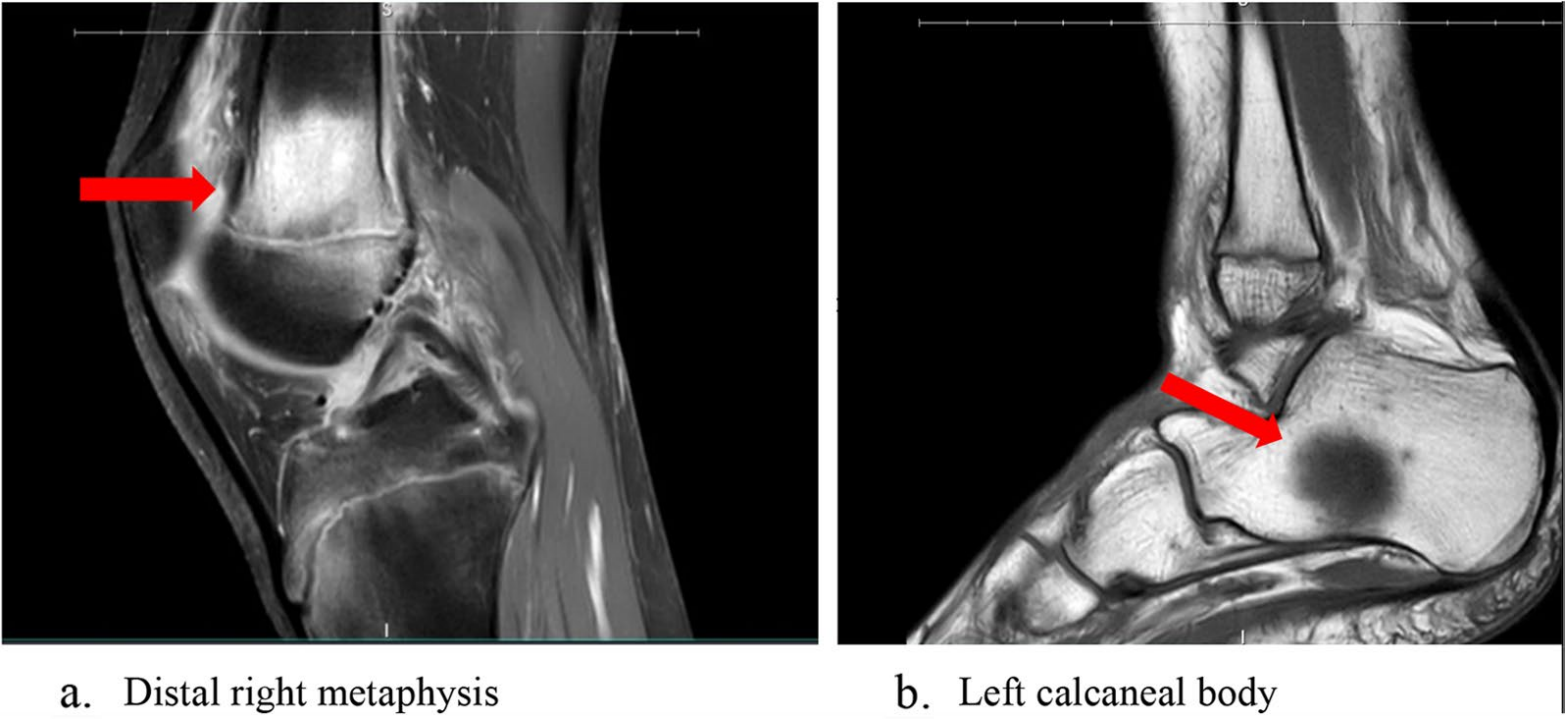
MRI imaging shows scattered bright T2 and dark T1, patchy enhancement of the distal right metaphysis (**a**) and the left calcaneal body (**b**)

Her perifollicular petechial hemorrhagic rash with corkscrew hairs was classic for scurvy, which was confirmed by dermatology. Unfortunately, vitamin C supplementation was started prior to obtaining baseline serum level. Despite knowing her findings were consistent with scurvy, we were concerned by the patient’s subsequent hospital course as she had recurrent symptomatic normocytic anemia (Hgb dropped from 7.1 g/dL on admission to 5.1 g/dL) requiring two blood transfusions, as well as recurrent fevers of unknown origin (39.7 °C). Hematology work up revealed a low iron level of 15 mcg/dL, and prolonged PT of 17.7 s and INR of 1.48. They attributed her anemia to iron deficiency, vitamin K deficiency, and anemia of chronic disease. Additional deficiencies included vitamin A, vitamin D, and folate, however vitamin B1 and vitamin B12 were normal. Inflammatory markers CRP and ESR were elevated at 30 mg/dL and 43 mm/hr respectively. Rheumatology serological workup was negative. Knee x-ray showed questionable osteopenia but no fractures. Chest x-ray showed mild scoliosis but otherwise no abnormalities. Neither x-ray showed any abnormalities of the growth plates. Ultrasound showing complex effusions was read by radiology as suggestive of septic arthritis, though this was disregarded as it was not clinically consistent. The effusions were not aspirated. Ankle and knee MRI showed patchy enhancement of the distal right metaphysis and calcaneal body with dark T1 and bright T2 signals (Fig. 3a, b).

Radiology reported the abnormalities on MRI as consistent with leukemia/lymphoma, chronic multifocal osteomyelitis, or Langerhans cell histiocytosis. Although malignancy was less likely due to the chronicity of her illness, due to her relatively recent deterioration (recurrent anemia, fevers, joint swelling) as well as our inability to explain the MRI abnormalities, the medical team including oncology decided a bone marrow biopsy was necessary. Bilateral knee bone marrow biopsy showed gelatinous transformation of the bone marrow (GBM), which is often seen in patients with malnutrition, especially in the setting of eating disorders [2]. After two weeks of nutrition management including vitamin C supplementation, the patient’s vitals normalized, and she was discharged to an inpatient eating disorder program. Ultimately, the patient’s presentation was explained by malnutrition and vitamin C deficiency secondary to ARFID.

## Discussion

Publications on pediatric scurvy have increased in recent years. These include diverse subspecialities including radiology, orthopedics, and developmental pediatrics. One developmental pediatrics review identified 20 reports involving 24 children with autism and ARFID who developed scurvy [3]. An institutional orthopedic review over nine years had nine patients [4]. A 2014 general pediatrics case study reviewed 10 separate cases [5]. Alten et al. reported their literature search identified 50 separate cases in the past 20 years, stating “it is no longer appropriate to view scurvy as a historical disease” [1]. Given this evidence, it is important to review scurvy’s diagnostic features.

Clinicians should be familiar with populations at risk for scurvy, including patients with eating disorders. In case reports of pediatric scurvy, patients usually had abnormal eating habits. Although many patients had autism and ARFID, several patients were otherwise developmentally normal yet still had ARFID or anorexia [6]. These patients, like our own, also had broad differentials including infectious, rheumatologic, and oncologic [5, 7, 8].

Clinicians should be familiar with both the common and uncommon or lesser-known presentations of scurvy. Our patient had classic features of scurvy including the characteristic perifollicular petechial hemorrhagic rash with corkscrew hairs, recurrent fevers, knee and ankle effusion, anemia, elevated inflammatory markers, and easy bruising [1, 4–7]. Only a few classic symptoms were not present, including, hemarthrosis (no aspirate was performed), gingival hemorrhages, dental losses, and active bleeding. Lesser-known features of scurvy include the abnormal radiographic findings**.** In our patient we were unfamiliar with the MRI features of scurvy when we decided upon bone marrow biopsy. In the radiology literature, there are reports of non-specific bone marrow changes associated with nutritional disorders including scurvy, anorexia nervosa, and GBM [9, 10]. Specifically, multiple scurvy cases report the same MRI abnormalities our patient had, including multifocal dark T1 and bright T2 signals [1, 8, 9, 11]. However, it also should be noted that, GBM may also rarely show bright bone marrow on T1-weighted and fluid sensitive images due to serous atrophy [9]. Interestingly, although her x-rays showed questionable osteopenia (osteopenia is often seen with scurvy), they did not have any other traditional hallmarks of scurvy such as scorbutic rosary or growth plate abnormalities [10].

## Conclusion

In conclusion, clinicians should keep scurvy on the differential of patients with malnutrition presenting with multisystem findings including anemia, fever, rash, joint swelling, or abnormal radiology findings. Specifically, clinicians who work with patients with eating disorders may need to have a heightened index of suspicion for scurvy. Importantly, the prevalence of scurvy in pediatric patients with malnutrition and/or eating disorders is currently unknown. Areas for future research include measuring the prevalence of scurvy in pediatric patients with malnutrition to determine if routine assessment of vitamin C in patients with eating disorder and/or malnutrition is justified.

## Patient statement

The patient recently was asked for a statement regarding her opinion on her care received. She commented mainly on her inpatient eating disorder facility stay, which was after the events of the case report. She stated “Not to say that the care I received was bad, but I felt like a lot my care wasn’t designed for someone with my eating disorder. A lot of it was around anorexia and body dysmorphia.” When asked how that made her feel she stated, “I felt like it wasn’t going to help me because it wasn’t dealing with the problems I had.” When asked if that was the case, she replied “Some of it did. Some of it didn’t.”

**Table:** 

Timeline of hospital stay	
Day	Notable events
1	Presented to emergency department. Normocytic anemia, tachycardia, abnormal orthostatic vital. X-ray bilateral knees showed possible osteopenia. Patient was admitted to general pediatrics ward. Adolescent Medicine consulted: diagnosed patient with ARFID and suspicion of scurvy. Started eating disorder management with nutrition and monitoring refeeding labs.
2	Febrile t-max 38.4 °C. Elevated CRP (30 mg/dL) and ESR (40 mm/h). Worsening anemia Hgb dropped from 7.1 to 5.1 g/dL required transfusion. Hematology Consulted: diagnosed patient with severe anemia secondary to iron, vitamin K and vitamin C deficiency and anemia of chronic disease. Folate, B1 and B12 levels normal. Vitamin D deficiency noted. Supplementation with iron, vitamin K and vitamin C started.
3	Knee ultrasound showed right knee joint effusion which may indicate septic arthritis. However, patient clinically not presenting with signs or symptoms of septic arthritis.
4–7	Infectious: recurrent fever, now t-max 37.8 °C, blood and urine cultures negative, HIV, treponemal antibody, Gonorrhea/chlamydia negative. Hematology: transfused a second time due to Hgb drop to 6.6 g/dL, further workup was negative. Homocysteine and methylmalonic acid levels were normal. Psychiatry consulted: mild depression and agreed with diagnosis of ARFID. Child abuse pediatrics consulted: no signs of abuse or neglect.
8–9	Rheumatology consulted: + ANA mildly positive 1:80, but inconsistent with JIA. Negative serologic workup. CK, ACE, Thyroglobulin, antimicrosomal TPO, complement, quantiferon gold, serum protein electrophoresis, RH, IL-6, Immunoglobulin levels, immune antibodies (Anti-Ds DNA, Anti-SM, Anti-RO, Anti-LA etc.). Rheumatology recommends knee and ankle MRI. MRI results are “Concerning for leukemia/lymphoma, however other differential considerations include chronic recurrent multifocal osteomyelitis (CRMO) and Langerhans cell histiocytosis”. Oncology consulted and recommended bone marrow biopsy. Orthopedic oncology consulted for biopsy later that week.
10–11	Dermatology consulted: agree rash is consistent with scurvy. Infectious: Continued fevers, t-max 39.7 °C. Oncology: chest x-ray and chest CT negative. Hematology: Coagulation studies are normalizing while on vitamin K supplementation.
12	Orthopedic oncology performs bilateral knee biopsy.
13–15	Continued management of eating disorder with nutrition. Weight improved and stable. Vitals normalized. Physical therapy obtained walker for patient. Patient defervesced. All cultures were negative. Discharged on day 15 with planned admission to inpatient eating disorder facility.

## Data Availability

Not applicable.

## Abbreviations

ARFID: Avoidant restrictive food intake disorder
GBM: Gelatinous transformation of bone marrow or gelatinous bone marrow

## References

[1] Alten ED, Chaturvedi A, Cullimore M, Fallon AA, Habben L, Hughes I (2020). No longer a historical ailment: two cases of childhood scurvy with recommendations for bone health providers. Osteoporos Int.

[2] Shergill KK, Shergill GS, Pillai HJ (2017). Gelatinous transformation of bone marrow: Rare or underdiagnosed?. Autops Case Rep.

[3] Sharp WG, Berry RC, Burrell L, Scahill L, McElhanon BO (2020). Scurvy as a sequela of avoidant-restrictive food intake disorder in autism: a systematic review. J Dev Behav Pediatr.

[4] Pan T, Hennrikus EF, Hennrikus WL (2020). Modern day scurvy in pediatric orthopaedics: a forgotten illness. J Pediatric Orthopaedics..

[5] Harknett KM, Hussain SK, Rogers MK, Patel NC (2013). Scurvy mimicking osteomyelitis. Clin Pediatr.

[6] Roy-Lavallee J, Bahrani B, Weinstein M, Katzman DK (2020). Scurvy: an unexpected nutritional complication in an adolescent female with anorexia nervosa. J Adolesc Health.

[7] Benezech S, Hartmann C, Morfin D, Bertrand Y, Domenech C (2020). Is it Leukemia, doctor? No, it’s scurvy induced by an ARFID!. Eur J Clin Nutr.

[8] Brennan CM, Atkins KA, Druzgal CH, Gaskin CM (2012). Magnetic resonance imaging appearance of scurvy with gelatinous bone marrow transformation. Skeletal Radiol.

[9] Chan BY, Gill KG, Rebsamen SL, Nguyen JC (2016). Mr imaging of pediatric bone marrow. Radiographics.

[10] Chang CY, Rosenthal DI, Mitchell DM, Handa A, Kattapuram SV, Huang AJ (2016). Imaging findings of metabolic bone disease. Radiographics.

[11] Ganske A, Kolbe AB, Thomas K, Hull N (2021). Pediatric scurvy MRI appearance. Radiol Case Rep.

